# Chiral Isochalcogenourea‐Catalysed Enantioselective (4+2) Cycloadditions of Allenoates

**DOI:** 10.1002/anie.202315345

**Published:** 2023-12-11

**Authors:** Lukas S. Vogl, Peter Mayer, Raphaël Robiette, Mario Waser

**Affiliations:** ^1^ Institute of Organic Chemistry Johannes Kepler University Linz Altenbergerstrasse 69 4040 Linz Austria; ^2^ Department Chemie Ludwig-Maximilians-Universität München Butenandtstraße 5–13 81377 München Germany; ^3^ Institute of Condensed Matter and Nanosciences Université catholique de Louvain Place Louis Pasteur 1 box L4.01.02 1348 Louvain-la-Neuve Belgium

**Keywords:** Allenoates, Cycloadditions, DFT Calculations, Lewis Bases, Organocatalysis

## Abstract

Allenoates are versatile building blocks which are primarily activated and controlled using chiral tert. phosphine and tert. amine Lewis bases. We herein report the first example of allenoate activation by using chiral isochalcogenoureas (IChU) for formal (4+2) cycloaddition reactions. Compared to established phosphine and amine catalysis, the use of these easily available Lewis bases enables new stereoselective reaction pathways proceeding with high enantioselectivities, diastereoselectivities, and in good yields. In addition, the factors governing enantioselectivity and the origin of the observed differences compared to other commonly used Lewis bases are explained.

## Graphical abstract

Chiral isochalcogenoureas (IChU) were successfully established as powerful organocatalysts for the activation and control of allenoates. This unprecedented strategy allows for unique reactivities and high levels of stereocontrol, facilitating reaction pathways that are not possible with the existing methods.

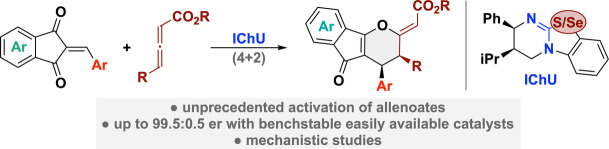

## Introduction

**Scheme 1:**
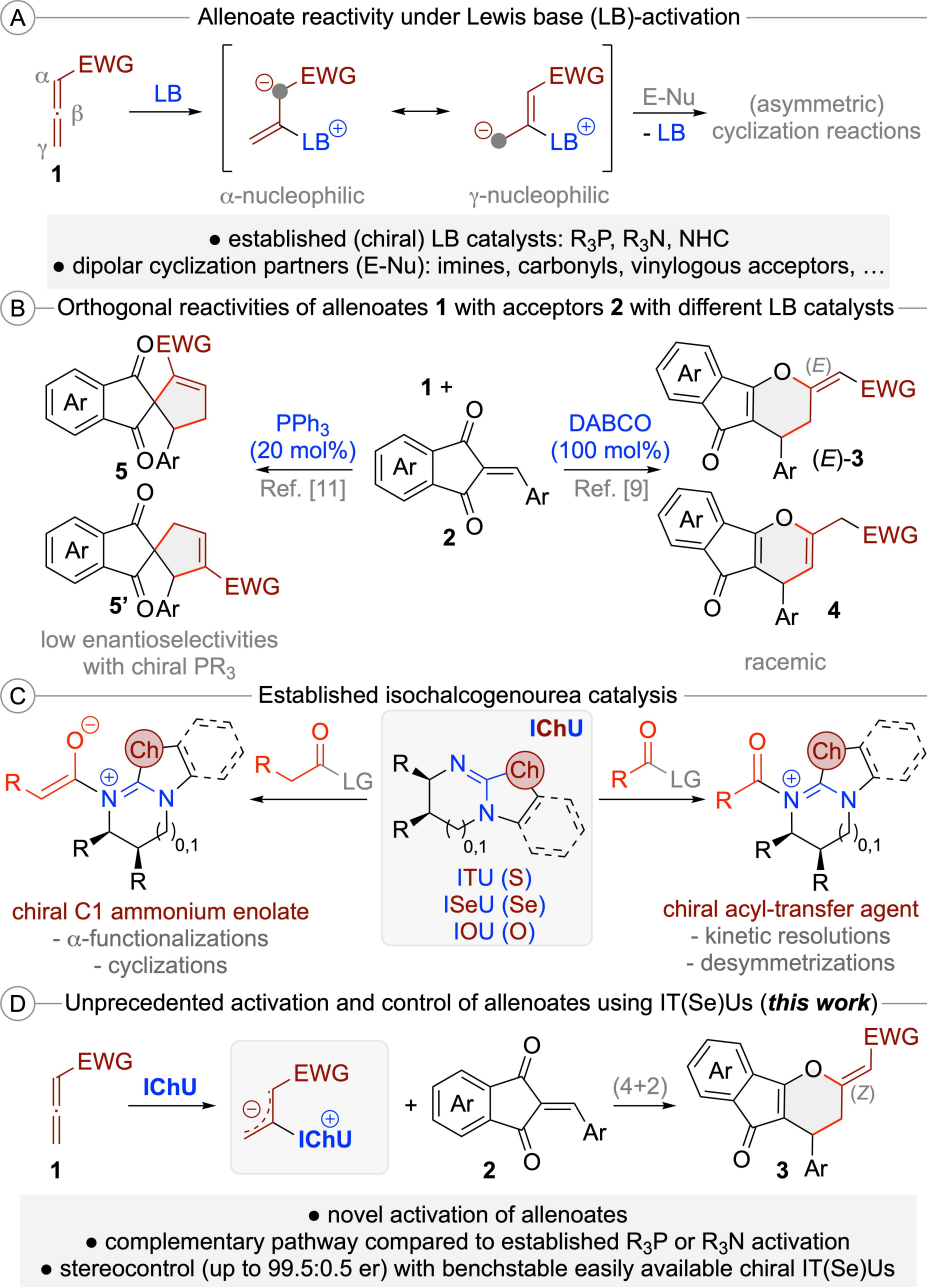
General activation of allenoates with Lewis bases (A); orthogonal reactivities of allenoates **1** with enones **2** using different Lewis bases (B); established applications of isochalcogenoureas (IChU) (C); and the herein reported novel activation of allenoates with isochalcogenoureas (D).

Allenoates **1** are easily accessible building blocks with extraordinary reactivity profiles, especially when activated using (chiral) Lewis base (LB) organocatalysts.[^1^, ^2^, ^3^, ^4^, ^5^] These nucleophilic catalysts activate and control the allenoate by adding to the sp‐hybridized β‐carbon, which results in the formation of reactive resonance stabilized betaine species (Scheme 1A). Depending on the used catalyst as well as the substitution pattern of the allenoate (in case further substituents in the α or γ‐position are present), either the α‐ or the γ‐carbon of these intermediates becomes the most nucleophilic position.[^1^, ^3^, ^4^, ^5^, ^6^] These zwitterionic species can react with a variety of dipolar molecules (E−Nu) via nucleophilic attack first, followed by release of the catalyst by “back‐biting” of the reaction partner, thus resulting in efficient structural complexity generating (stereoselective) formal cycloaddition reactions (Scheme 1A).

With respect to the catalyst motives of choice, the field is clearly dominated by (chiral) tertiary phosphines,^[3]^ but tertiary amines^[4]^ and *N‐*heterocyclic carbenes^[5]^ have been successfully utilized too. Very importantly, the nature of the used catalyst has a pronounced effect on these reactions, which often allows for orthogonal reactivities and connectivities when utilizing different catalyst principles,^[6]^ thus allowing for diversity‐oriented approaches as well.^[7]^ A considerable challenge, however, is the fact that chiral catalyst derivatives occasionally show lower reactivities than achiral ones, making identification of a suited chiral catalyst potentially difficult.^[8]^ In addition, sometimes higher catalyst loadings (>10 mol %) are necessary, which is to some extent due to the possible sensitivity of P(III)‐based catalysts towards oxidation.

An example that nicely illustrates the potential, but also these challenges, is the cycloaddition of allenoates **1** with the reactive Michael acceptors **2** (Scheme 1B).[^9^, ^10^, ^11^] In the presence of DABCO, this reaction yields the formal (4+2) cycloaddition products (*E*)‐**3** and **4**, whereby the location of the double bond depends on the reaction conditions.^[9]^ Unfortunately, this combination required a stoichiometric amount of the amine and no asymmetric variant has been described. In contrast, the use of PPh_3_ gives the regioisomeric (3+2) cycloaddition products **5**.^[11]^ In this case a first asymmetric proof‐of‐concept using chiral phosphines with low enantioselectivities was obtained, albeit leaving space for improvement.^[11b]^ Accordingly, the establishment of alternative (chiral) catalyst motives for allenoate transformations could be a highly rewarding task. With a suited complementary catalyst platform at hand, it should be possible to enter reaction pathways that are not possible with the existing catalysts, thus providing access to orthogonally functionalized product scaffolds, even in an asymmetric manner.

One class of highly valuable and easily accessible bench stable chiral LB organocatalysts^[2]^ are isochalcogenoureas (IChU), i.e. well‐established isothioureas (ITU)[^12^, ^13^, ^14^] and the more recently introduced Se‐ and O‐analogs.^[15]^ While these well‐understood nucleophilic catalysts^[14]^ have been very frequently used for asymmetric acyl‐transfer reactions as well as C1 ammonium enolate reactions (Scheme 1C),^[16]^ to the best of our knowledge there have been no reports describing their use for (asymmetric) allenoate transformations so far. We thus started a program focusing on the use of IChU as Lewis base catalysts to activate and control reactions of allenoates **1** with Michael acceptors **2** (Scheme 1D). These reactive enones were chosen because of their well‐documented high reactivity^[17]^ and because of the afore mentioned fact that they have already been reported as allenoate‐cycloaddition partners with established phosphine and amine‐catalysts (Scheme 1B).[^9^, ^10^, ^11^] Thus, we were hoping that the unprecedented use of IChU will allow for alternative cycloaddition pathways, even in a stereocontrolled manner. In addition, potential products like the spirocyclic compounds **5** or the (dihydro)pyrane‐containing **3** and **4** represent interesting targets because of the well‐described biological properties associated to these important scaffolds (which are also frequently found in natural products).[^18^, ^19^] In this manuscript we demonstrate for the first time that allenoate‐activation using IT(Se)U catalysis is indeed possible and allows for formal (4+2) cycloadditions yielding dihydropyrane derivatives (*Z*)‐**3** in a highly enantioselective fashion by using these easily accessible bench stable chiral Lewis base organocatalysts. In addition, the factors governing enantioselectivity and the origin of the observed differences in reactivity between tertiary phosphine‐, DABCO‐ and IChU‐catalysed reactions are also explained.

## Results and Discussion

### Optimization of Reaction Conditions

We started our investigations by testing the reaction between the ethyl allenoate **1 a** and the parent acceptor **2 a** using the well‐established isochalcogenoureas **ITU1**‐**5** and the previously^[15]^ introduced **ISeU** and **IOU** (Table 1). First experiments using achiral **ITU1** at room temperature in different solvents and with various bases (as exemplified in entry 1) were discouraging and resulted in no reaction between **1 a** and **2 a** at all (for comparison: PPh_3_ and DABCO promote these reactions at r.t.[^9^, ^10^, ^11^]). Interestingly however, when analysing the crude reaction mixtures by ESI‐MS, a species corresponding to a potential adduct of **1 a** and **2 a** was observed. In addition, we also observed a species with m/z=303.1168 in the positive ion mode, which corresponds to a protonated catalyst‐allenoate adduct like **int‐A**. These ESI‐MS observations suggested that higher temperatures (as present in the ESI device) may be required to facilitate the initial addition of **ITU1** to **1 a** and subsequent reactions of **int‐A** with **2 a**. Gratifyingly, when carrying out the reaction at 80 °C (entry 2), a distinct addition product was isolated, which, upon careful structural analysis, could be identified as (*Z*)‐**3 a** (no reaction takes place at elevated temperature in the absence of catalyst; entry 3). Formation of this product can be explained by γ‐addition of **int‐A** to the enone **2 a** followed by O‐back‐attack via the β‐carbon of the allenoate and elimination of the catalyst, which also sets the double bond configuration (see below). Interestingly, in these first experiments we also observed smaller amounts of the α‐addition product **6 a**,^[20]^ but only traces (less than 5 %) of the (*E*)‐diastereoisomer, which is the favoured one under the DABCO‐mediated conditions.^[9]^ With this encouraging proof‐of‐concept at hand, which underscores the complementary nature of this ITU‐catalysed approach compared to established amine‐ or phosphine‐catalysed ones, we next screened base and solvent (entries 2–9) as well as different stoichiometric ratios of reagents and base. Non‐aromatic polar solvents gave the product in significantly reduced yields and resulted in lower conversions and larger amounts of by‐products (entries 8 and 9). Base‐free toluene conditions were tolerated too (entry 7), but with a slightly lower yield as compared to the base‐mediated ones (entries 2 vs 7) and we also found the reaction being more robust and reliable in the presence of base (the reason may be that the base traps traces of eventually present acids which can have a detrimental effect on the reaction—see below). Overall we thus identified 1 eq. K_2_CO_3_ in toluene with 1 eq. of **2 a** and a slight excess of **1 a** (1.5 eq.) to be best‐suited to access *rac*‐(*Z*)‐**3 a** in a good isolated yield of 74 % (entry 2).

**Table 1:** Screening and optimization of the IT(Se)U‐catalysed reaction of **1 a** with **2 a**.^[a]^

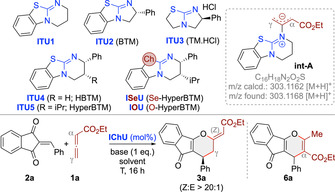							
Entry	IChU (mol %)	Solv.	Base	T [°C]	**3 a** [%]^[b]^	er^[c]^	**6 a** ^[d]^ [%]
1	**ITU1** (20)	toluene	K_2_CO_3_	25	n. r.	–	–
2	**ITU1** (20)	toluene	K_2_CO_3_	80	74	50 : 50	15
3	–	toluene	K_2_CO_3_	80	n. r.	–	–
4	**ITU1** (20)	toluene	Cs_2_CO_3_	80	40	50 : 50	15
5	**ITU1** (20)	toluene	K_3_PO_4_	80	61	50 : 50	20
6	**ITU1** (20)	toluene	DIPEA	80	65	50 : 50	20
7	**ITU1** (20)	toluene	–	80	69	50 : 50	15
8	**ITU1** (20)	CH_2_Cl_2_	–	60	34	50 : 50	20
9	**ITU1** (20)	THF	–	60	10	50 : 50	–
10	**ITU2** (20)	toluene	K_2_CO_3_	80	n.r.	–	–
11	**ITU3** (20)	toluene	K_2_CO_3_	80	n.r.	–	–
12	**ITU4** (20)	toluene	K_2_CO_3_	80	79	92 : 8	5
13	**ITU5** (20)	toluene	K_2_CO_3_	80	77	94 : 6	<5
14	**ITU5** (20)	toluene+H_2_O (1x)	K_2_CO_3_	80	56	94 : 6	<5
15	**ITU5** (20)	toluene	K_2_CO_3_	60	75	93 : 7	<5
16	**ITU5** (20)	toluene	K_2_CO_3_	40	59	90 : 10	5
17	**ITU5** (5)	toluene	K_2_CO_3_	80	78	95 : 5	<5
18	**ITU5** (5)	toluene^[e]^	K_2_CO_3_	80	69	91 : 9	<5
19	**ISeU** (5)	toluene	K_2_CO_3_	80	83	97 : 3	<5
20	**ISeU** (5)	toluene	K_2_CO_3_	80	88^[f]^	98 : 2^[f]^	<5
21	**IOU** (5)	toluene	K_2_CO_3_	80	36	84 : 16	<5
22	**ISeU** (1)	toluene	K_2_CO_3_	80	82	97 : 3	<5
23	**ISeU** (0.1)	toluene	K_2_CO_3_	80	16	96 : 4	<5

[a] All reactions were run for 16 h using 0.1 mmol **2 a**, 0.15 mmol **1 a**, 0.1 mmol of the base, and the indicated IChU in the given solvent (0.02 M based on **2 a**) unless otherwise stated. [b] Isolated yield of (*Z*)‐**3 a**. [c] Determined by HPLC using a chiral stationary phase. The absolute configuration was assigned in analogy to derivative **3 p** (see below). [d] Amount calculated from the ^1^H NMR of the crude reaction mixture. [e] 0.05 M [f] 1 mmol scale.—n.r. means no reaction.

With reliable high yielding conditions for the non‐enantioselective approach at hand (K_2_CO_3_, toluene, entry 2), we next focused on the asymmetric variant by using chiral **ITU2**‐**5** (entries 10–13). Interestingly, the bicyclo[3.3.0]octene‐based **ITU2** and **ITU3** did not allow for any product formation at all (entries 10, 11). In sharp contrast, the bicyclo[4.3.0]octene‐based **ITU4** and **ITU5** gave (*Z*)‐**3 a** with high enantioselectivities and in even higher yields and with significantly smaller amounts of α‐addition product **6 a** as compared to achiral **ITU1** (entries 12,13 vs 2). The better performance observed with **ITU4** and **ITU5** as compared to the smaller ring derivatives **ITU2** and **ITU3** may be attributed to their higher nucleophilicity^[14]^ and points towards a mechanistic scenario where the addition of the IChU to the allenoate might be the rate limiting step (see below). Remarkably, the high reaction temperatures that are necessary for the cyclization to proceed allowed for high enantioselectivities up to 94 : 6 with **ITU5** in these first experiments already. The presence of H_2_O resulted in a reduced yield and conversion but without affecting e.r. (entry 14; addition of molecular sieves did not influence the reaction at all), thus underscoring the need for H_2_O‐free solvents and reagents. Attempts to increase the er by lowering the temperature did not improve the outcome (entries 15 and 16; higher T was not beneficial either). Based on the discouraging results when using more polar solvents for the reaction leading to racemic mixtures (entries 8 and 9), no systematic screening of other solvents for the asymmetric protocol was carried out anymore (a test reaction with **ITU5** in THF however showed that only traces of product **3 a** were formed in this case (result not given in the Table)). Interestingly, lowering the amount of catalyst to 5 mol % lead to a slightly improved er (entry 17) and allowed for a rather clean reaction, while carrying out the reaction under more concentrated conditions (entry 18) reduced selectivity and yield. Finally, we also tested the less‐commonly used **ISeU** and **IOU** (entries 19–23).^[15]^ While **ISeU** was recently very successfully used for acyl‐transfer reactions as well as C1 ammonium enolate transformations,[^15^, ^16l^] **IOU** was reported to be catalytically less competent than the S‐ or Se‐analogs.^[15]^ The same trend was also observed in our case: While **IOU** gave product **3 a** in low yield and with significantly reduced enantioselectivity (entry 21), **ISeU** allowed for high yield (88 %) and enantioselectivity (98 : 2 er) even on 1 mmol scale (entry 20) and when reduced to 1 mol % catalyst loading (entry 22; lower catalyst loadings as outlined in entry 23 resulted in reduced conversions and yields but still with high er).

### Application Scope and Further Manipulations

**Scheme 2:**
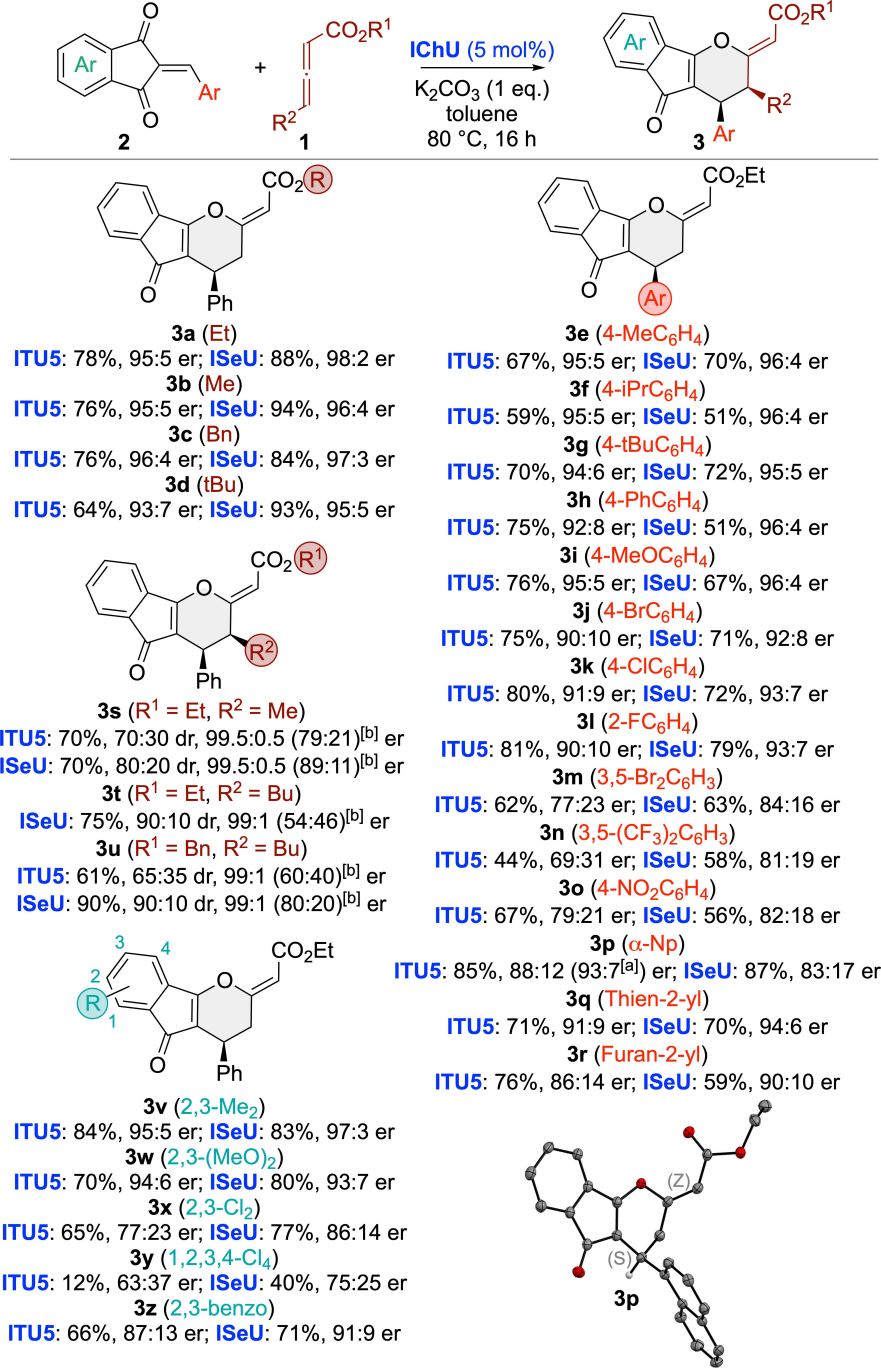
Asymmetric application scope of the (4+2) cycloaddition (isolated yields; [a] er after recrystallization; [b] er of the minor diastereoisomers). Molecular structure of **3 p** determined by single‐crystal X‐ray structure analysis.

Having identified the best‐suited asymmetric reaction conditions, we next investigated the application scope for a variety of differently functionalized acceptors **2** and allenoates **1** (Scheme 2). To allow for robust conditions and comparable results we systematically used 5 mol % of **ITU5** and **ISeU**. In most cases **ISeU** was found to be slightly more selective and the reaction was found to be pretty tolerant to various modifications on both reagents. While allenoate ester modifications (**3 a**–**3 d**) were all equally well‐accepted, the nature, i.e. the electronic properties, of different arylidene groups (**3 e**–**3 r**) had a stronger effect, and especially electron‐poor aryl groups lead to lower selectivities as outlined for **3 n** and **3 o**. Gratifyingly the Np‐containing **3 p** gave crystals suited for X‐ray diffraction analysis^[21]^ which allowed us to unambiguously confirm the double bond configuration being *Z* and the absolute configuration being *S*.

Noteworthy, γ‐substituted allenoates were also very well‐tolerated, giving the γ‐addition products **3 s**–**3 u** with good yields and diastereoselectivities (favouring the *cis*‐substituted isomer) and excellent enantioselectivities (interestingly **3 t** was the only case where no reaction took place when using **ITU5**). Finally, variations of the indandione part were also tested (**3 v**–**3 z**). Here we finally approached the limits of this methodology as electron‐poorer acceptors like **3 y** reacted significantly slower and also gave lower selectivities as compared to electron‐richer ones like **3 v** or **3 w**.

To get a deeper understanding on the functional group tolerance we also carried out a short robustness test by carrying out the non‐selective synthesis of product **3 e** (with **ITU1**) in the presence of phenol, isobutanol, benzoic acid, aniline, *N*‐benzylbenzamide and benzaldehyde.^[22]^ While butanol, aniline, and *N*‐benzylbenzamide showed very little effect, the presence of stronger acids such as phenol and benzoic acid significantly lowered the product yield (details can be found in the SI). These results, which can be rationalized by a detrimental protonation (and thus quenching of reactivity) of the intermediate betaine species, underscore the need for aprotic conditions (compare with entry 14, Table 1) and the beneficial effect of the present base. A lower yield was also observed in the presence of benzaldehyde which can be rationalized by addition of the activated allenoate to the benzaldehyde.

**Scheme 3:**
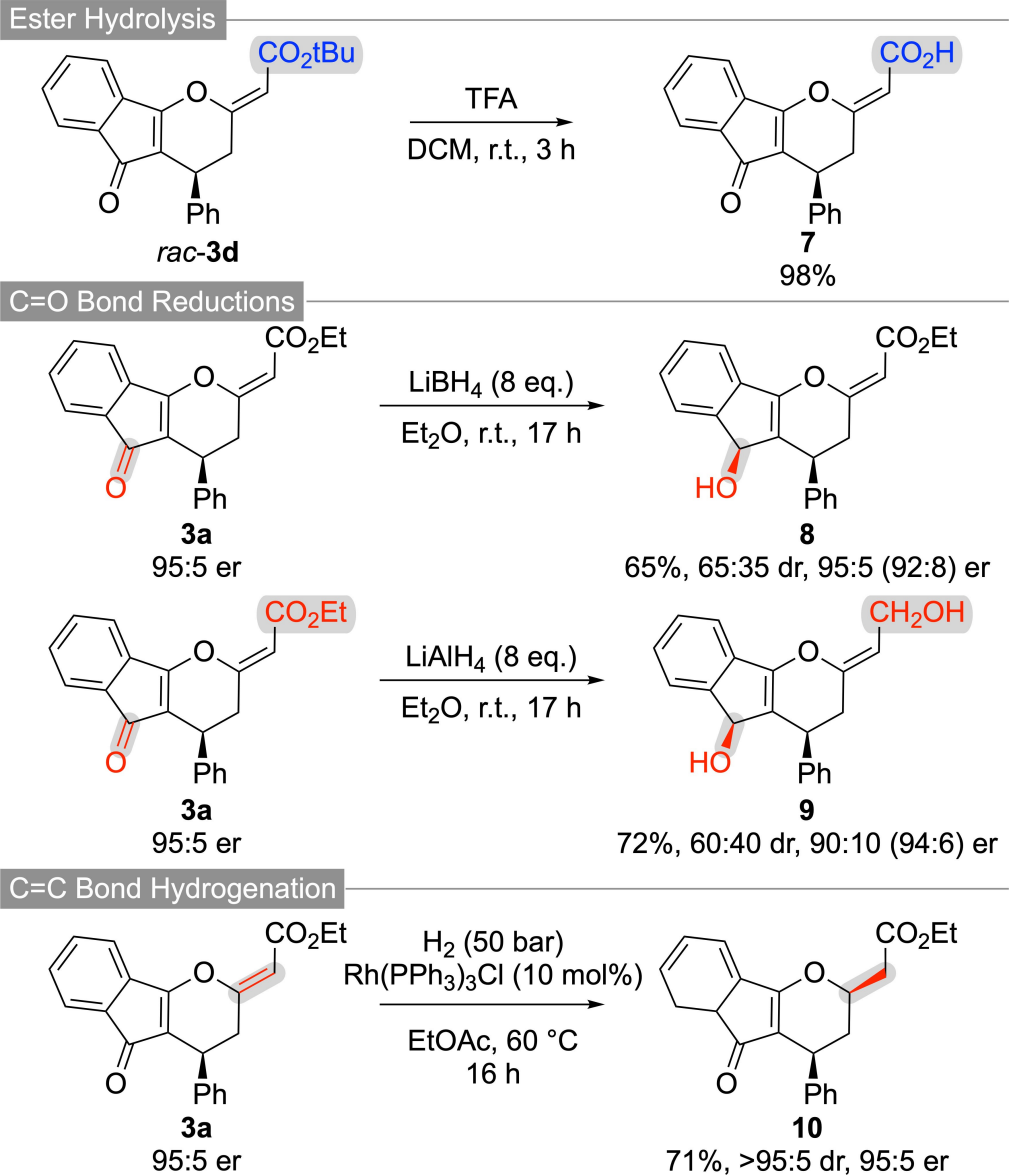
Further functionalizations of compounds **3**.

To demonstrate the synthetic value of the highly functionalized tricyclic compounds **3**, several follow‐up transformations have been carried out. As outlined in Scheme 3, the t‐butyl ester of **3 d** can easily be hydrolysed under acidic conditions giving the free acid **7**. In addition, various reductions either utilizing complex hydrides or by using transition metal‐catalysed hydrogenations are possible. While LiBH_4_ allows for the chemoselective ketone reduction (giving **8** as a mixture of diastereoisomers), LiAlH_4_ reduces both, the ketone and the ester functionality (product **9**). In both cases some erosion of er can be observed, but it should be emphasized that no further optimization addressing these subtle levels of epimerization has been carried out anymore. Alternatively, a transition metal‐catalysed homogeneous hydrogenation using Wilkinson's catalyst allowed for the selective reduction of the exocyclic C−C double yielding product **10** with high diastereoselectivity.

### Mechanistic Investigations

**Figure 1:**
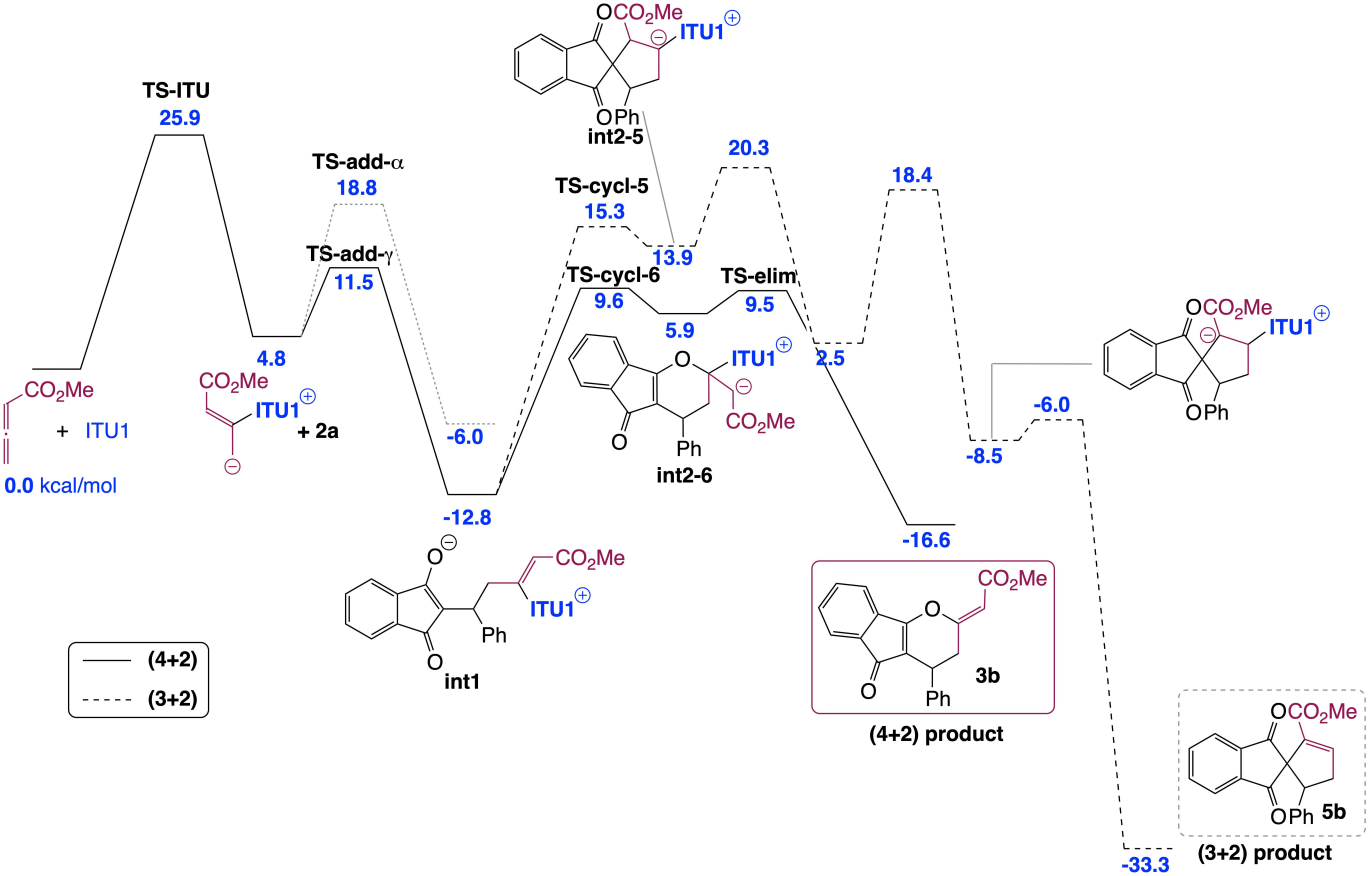
Computed free energy profile (kcal/mol relative to reactants) for the formation of **3 b** and **5 b** (see Supporting Information for additional data and full details).

To get a fundamental understanding of the mechanism and the factors controlling the regio‐, diastereo‐ and enantioselectivities in the IChU‐catalysed (4+2) cycloaddition of allenoates, we have examined the free energy profile of the reaction between methyl allenoate **1 b** and **2 a** catalysed by **ITU1** (Figure 1). The calculations were carried out at the M06‐2X/6–311+G**//M06‐2X/6‐31G* level of theory, with a continuum description of toluene as the solvent (see Supporting Information for full details).^[23]^ All the potential mechanisms for the **ITU1**‐catalysed reaction between **1 b** and **2 a** were investigated (see Supporting Information for full details). The predicted most favoured pathways for the (4+2) and (3+2) cycloaddition processes are depicted in Figure 1.

Our calculations indicate that the reaction begins by the addition of the catalyst onto the allene, in good agreement with the experimental observation of **int‐A** (see Table 1). As suggested by experiment (see above), this first step is computed to be the rate determining step of the reaction, with a free energy barrier of 25.9 kcal/mol, explaining the high temperature necessary for the reaction to proceed. The catalyst‐allenoate adduct can then add onto the Michael acceptor (**2 a**) according to two different modes: α‐ or γ‐addition. For steric reasons, the γ‐addition is predicted to be much more favourable, proceeding through a low free energy barrier (6.7 kcal/mol) to form **int1**. This latter can then cyclize toward a five‐ or a six‐membered ring to lead to a (3+2) or a (4+2) cycloaddition product (**5 b** or **3 b**, respectively). The observed exclusive formation of **3 b** can be explained by the high endergonicity of the five‐membered ring formation and the subsequent high free energy barrier to proton transfer.

**Scheme 4:**
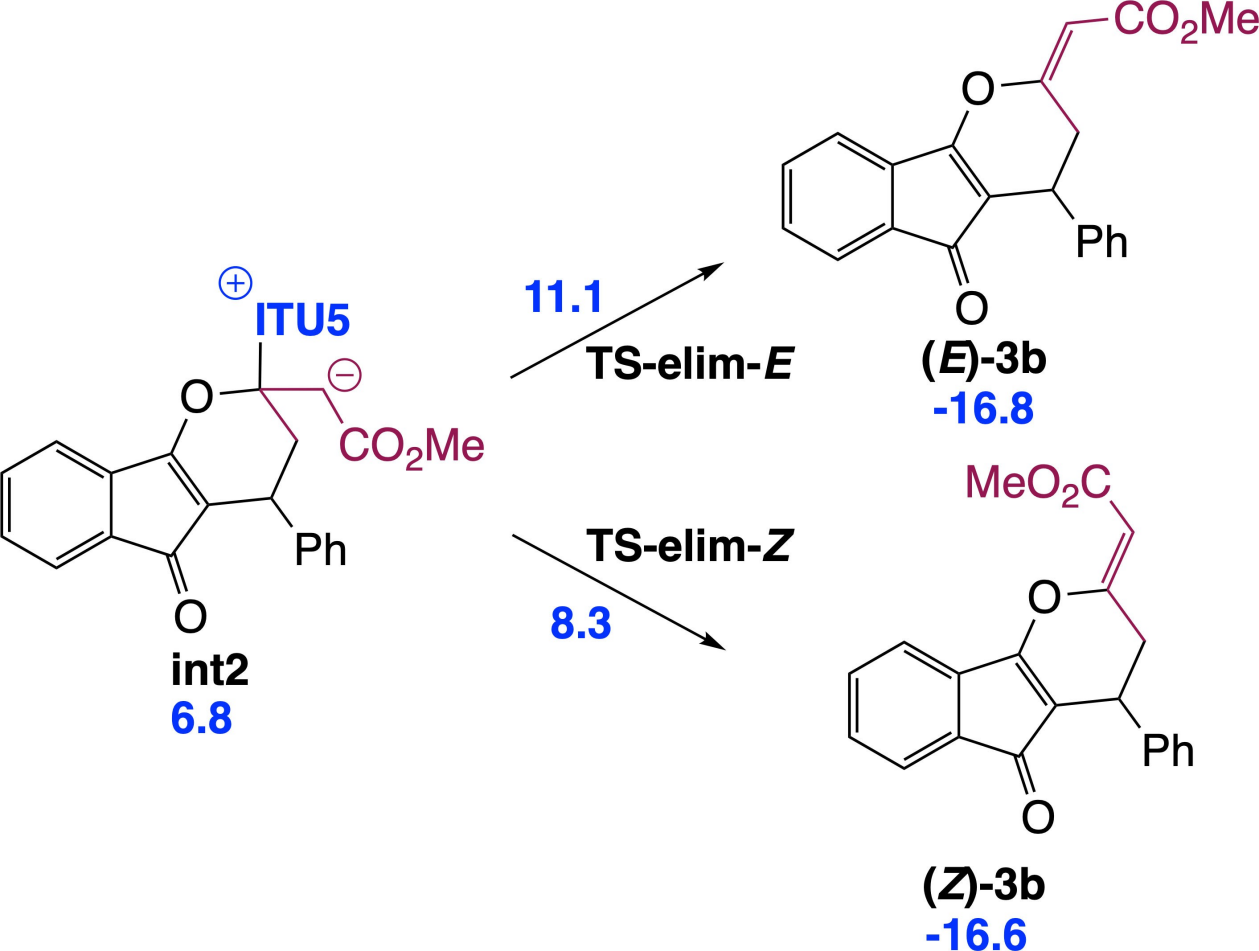
Origin of the high Z selectivity of the exocyclic double bond formed in the IChU‐catalysed (4+2) cycloaddition (relative free energy to reactants in kcal/mol).

Our IChU‐catalysed (4+2) cycloaddition reaction provides dihydropyranes **3** with an excellent (>20/1) *Z/E* selectivity in favour of the *Z* isomer. According to the mechanism, double bond configuration is set in the last step, the elimination of the catalyst (**int2**→**3**). Computed free energy of the two transition states (**TS‐elim‐*E* and TS‐elim‐*Z*)** predicts a high selectivity in favour of the *Z* isomer, in good agreement with experiment (Scheme 4).

**Scheme 5:**
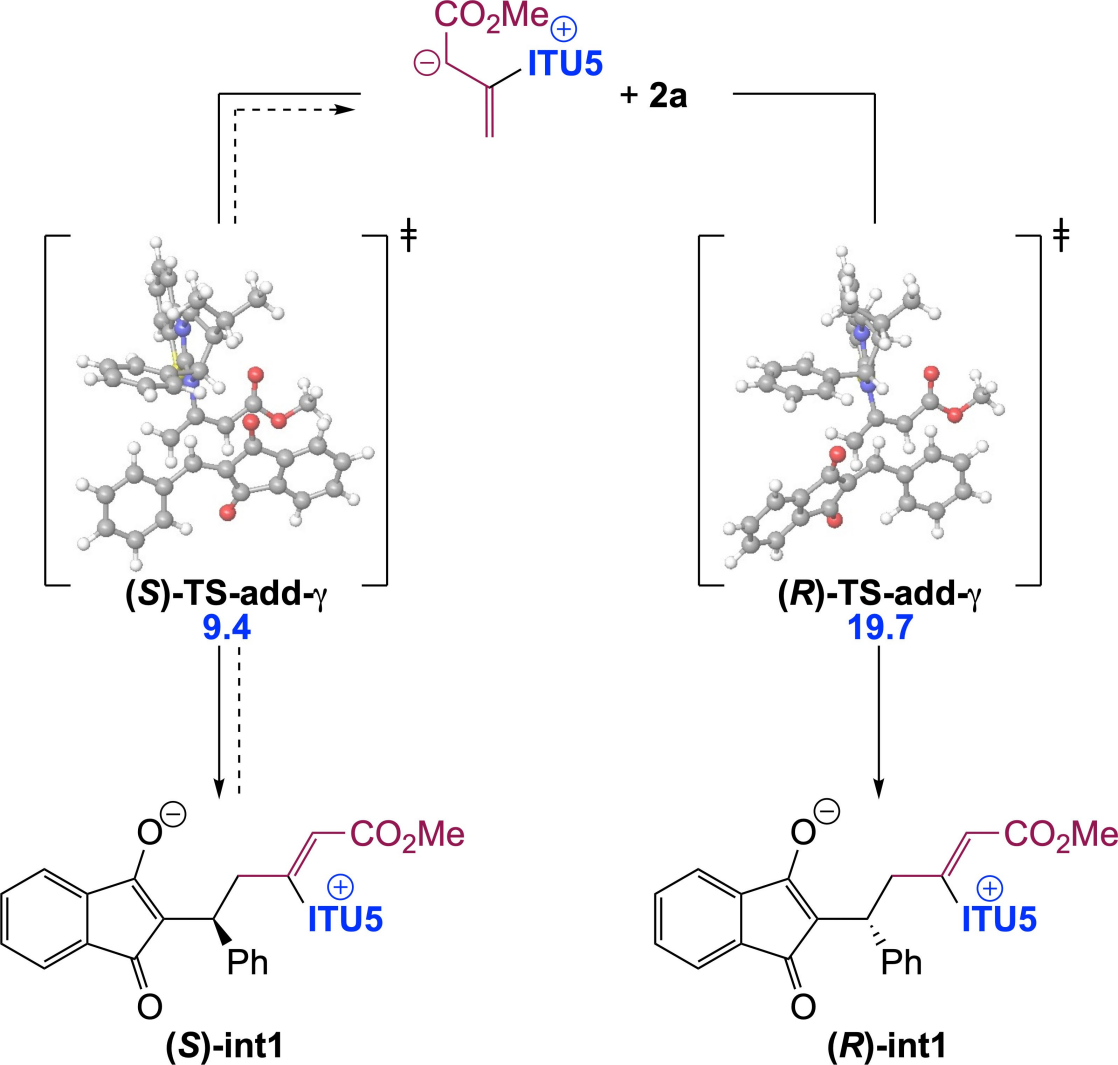
Origin of enantioselectivity in the chiral IChU catalysed (4+2) cycloaddition (relative free energy to reactants in kcal/mol).

The stereogenic centre of dihydropyranes **3** is set during the addition step, leading to **int1**. Our calculations indicate that this step is essentially non‐reversible. Accordingly, in order to get insights into factors controlling enantioselectivity, we investigated the two diastereomeric transition states for addition (**(*S)−*TS‐add‐γ** and **(*R)−*TS‐add‐γ**) in the case of HyperBTM (**ITU5**) as the chiral catalyst (Scheme 5).

Obtained relative free energies show a large discrimination between the two faces of the Michael acceptor (>10 kcal/mol difference), predicting >99 : 1 er. Experimentally observed enantioselectivities (95 : 5 er for (S)‐**3 a**, Scheme 2) are in good but not complete agreement with this computed selectivity. This could be accounted for by a partial reversibility of the addition step which would enable the epimerization of **int1**. Indeed, the free energy difference between **(*S)−*TS‐add‐γ** and the subsequent **(*S)−*TS‐cycl‐6** (compare with Figure 1) is 1.0 kcal/mol (for **ITU5**). The main factor determining enantioselectivity in our IChU‐catalysed (4+2) cycloaddition methodology is thus not only the facial selectivity but also the degree of reversibility of the addition step.

This finding provides an explanation for the observed decrease in enantioselectivity when the Michael acceptor (**2**) is substituted by electron‐withdrawing groups (see **3 m**–**o**, **3 x** and **3 y** in Scheme 2). Indeed, in these cases, the increased electrophilicity of **2** leads to a lowering of the free energy barrier to addition (**TS‐add‐γ**), without impacting significantly the cyclization step (**TS‐cycl‐6**), what favours the reversibility of addition, and hence epimerization of the stereogenic centre.

**Figure 2:**
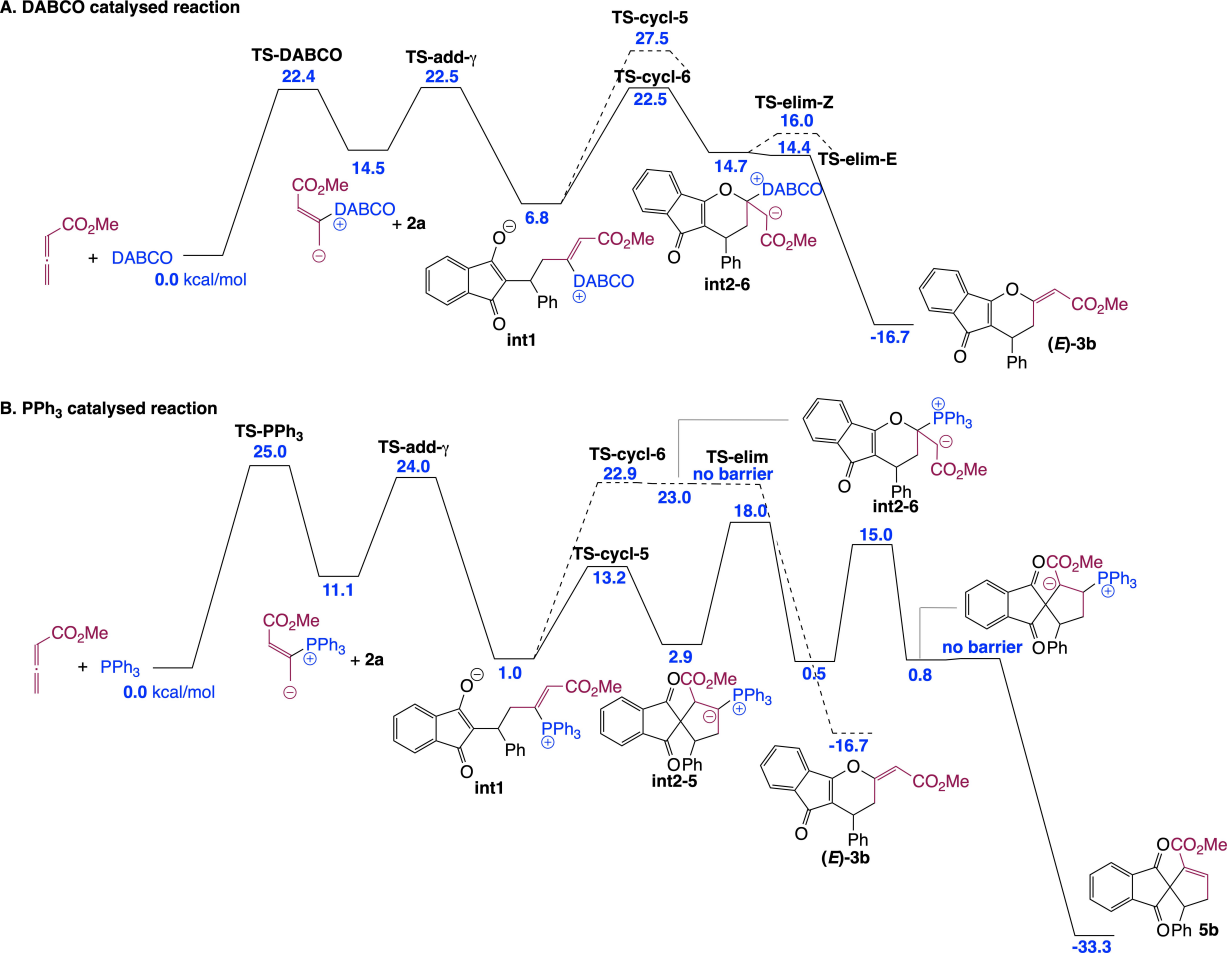
Computed free energy profile (kcal/mol relative to reactants) for the complementary cycloadditions catalysed by DABCO (A) and PPh_3_ (B) (see Supporting Information for additional data and full details).

Next, we sought to understand the origin of the difference in reaction product between PPh_3_‐, DABCO‐ and IChU‐catalysed cycloadditions of allenoates (see Scheme 1B and 1D). The computed free energy profile for the DABCO‐catalysed process indicates that formation of the catalyst‐allene adduct is more endergonic than with IChU and the subsequent addition onto **2** is also less endergonic (Figure 2). It results that intermediate **int1** lies much higher in energy than in the case of the IChU‐catalysed reaction and the three key transition states (**TS‐DABCO**, **TS‐add‐γ** and **TS‐cycl‐6**) have the same free energy, thus indicating a possible (partial) reversibility of the two first steps. In terms of *E/Z* selectivity, computed free energy of **TS‐elim‐*E*** and **TS‐elim‐*Z*** is 14.4 and 16.0 kcal/mol, respectively, predicting now a selectivity in favour of the *E* isomer, in good agreement with experiment (see Scheme 1B).^[9]^

In the case of the PPh_3_‐catalysed process, the addition of the catalyst onto the allene and the subsequent reaction with the Michael acceptor are similar to that with DABCO. However, for the cyclization step, there is a high preference for the formation a five‐membered ring, in the case of PPh_3_. This is due to the high stabilization of the negative charge by the phosphonium group in **int2**‐**5** (cyclization toward a six‐membered ring does not lead to a phosphonium ylide but to an enolate, see **int2**‐**6** in Figure 2B). It results that the (3+2) cycloaddition reaction is favoured over the (4+2) cycloaddition when PPh_3_ is used as a catalyst, in good agreement with reported experimental data.^[11]^

**Scheme 6:**
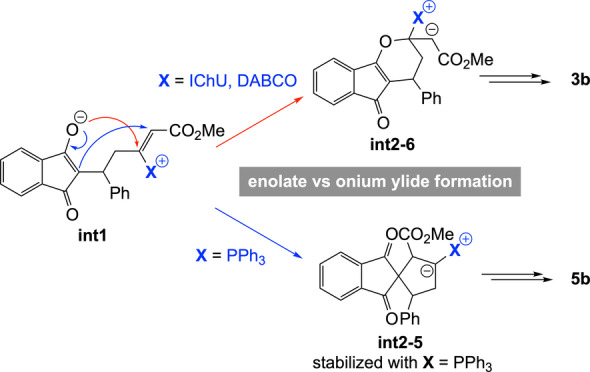
Rationale for the orthogonal reactivities ((4+2) vs (3+2)) between amine‐ (IChU or DABCO) and phosphine‐catalysed cycloadditions.

Overall, the observed orthogonal reactivities between amine‐ (IChU or DABCO) and phosphine‐catalysed cycloadditions can be accounted for by the high electrophilicity of the vinylphosphonium function, which outperform the one of the acrylate moiety and hence favour a 5‐membered ring cyclization, whereas vinylammonium species are less electrophilic, leading to a 6‐membered ring cyclization by O‐1,4‐addition of the enolate onto the acrylate group (Scheme 6).

## Conclusion

We succeeded in establishing the so far unprecedented use of chiral isochalcogenoureas as efficient Lewis base organocatalysts for the activation of allenoates, resulting in a versatile approach for asymmetric cycloaddition reactions that are not possible with the established catalysts. More specifically, allenoates **1**, which have so far mainly been employed in combination with tert. phosphine or tert. amine catalysis, were successfully used for asymmetric formal (4+2) cycloadditions with activated Michael acceptors **2**. This combination allows for alternative reaction pathways compared to the established catalysts by giving the highly functionalized products **3** with high levels of stereocontrol and in high yields. Besides establishing scope and limitations and demonstrating the value of these products for further manipulations, we also elucidated the factors governing enantioselectivity and the origin of the observed differences compared to other commonly used Lewis bases. We are convinced that the herein established introduction of isochalcogenoureas as versatile catalysts for asymmetric allenoate reactions holds much promise for future applications in the broad field of allenoate chemistry. This concept opens the door towards alternative reaction pathways compared to established catalysis principles and it also allows for high levels of stereocontrol by using low quantities of easily accessible and bench stable catalysts. Further studies investigating the generality of this (4+2) cycloaddition by using other classes of acceptors are ongoing and the results will be reported in due course.

## Supporting Information

The authors have cited additional references within the Supporting Information.[^24^, ^25^, ^26^, ^27^]

## Conflict of interest

The authors declare no conflict of interest.

1

## Supporting information

As a service to our authors and readers, this journal provides supporting information supplied by the authors. Such materials are peer reviewed and may be re‐organized for online delivery, but are not copy‐edited or typeset. Technical support issues arising from supporting information (other than missing files) should be addressed to the authors.

Supporting Information

## Data Availability

The data that support the findings of this study are available in the supplementary material of this article.
